# Examining Individual Differences in Language Learning: A Neurocognitive Model of Language Aptitude

**DOI:** 10.1162/nol_a_00042

**Published:** 2021-08-20

**Authors:** Sabrina Turker, Annemarie Seither-Preisler, Susanne Maria Reiterer

**Affiliations:** Lise Meitner Research Group Cognition and Plasticity, Max Planck Institute for Human Cognitive and Brain Sciences, Leipzig, Germany; Centre for Systematic Musicology, University of Graz, Graz, Austria; BioTechMed Graz, Graz, Austria; Department of Linguistics, University of Vienna, Vienna, Austria

**Keywords:** individual differences, language learning, neuroimaging, neurobiology, arcuate fascicle

## Abstract

A common practice in the cognitive neurosciences is to investigate population-typical phenomena, treating individuals as equal except for a few outliers that are usually discarded from analyses or that disappear on group-level patterns. Only a few studies to date have captured the heterogeneity of language processing across individuals as so-called “individual differences”; fewer have explicitly researched language aptitude, which designates an individual’s ability for acquiring foreign languages. Existing studies show that, relative to average learners, very gifted language learners display different task-related patterns of functional activation and connectivity during linguistic tasks, and structural differences in white and grey matter morphology, and in white matter connectivity. Despite growing interest in language aptitude, there is no recent comprehensive review, nor a theoretical model to date that includes the neural level. To fill this gap, we review neuroscientific research on individual differences in language learning and language aptitude and present a first, preliminary neurocognitive model of language aptitude. We suggest that language aptitude could arise from an advantageous neurocognitive profile, which leads to high intrinsic motivation and proactive engagement in language learning activities. On the neural level, interindividual differences in the morphology of the bilateral auditory cortex constrain individual neural plasticity, as is evident in the speed and efficiency of language learning. We suggest that language learning success is further dependent upon highly efficient auditory-motor connections (speech-motor networks) and the structural characteristics of dorsal and ventral fibre tracts during language learning.

## INTRODUCTION

Among the numerous social, cognitive, and affective variables that influence second language learning success (see Ellis, 2004, for an overview), research suggests that motivation, age of onset, and language aptitude are by far the most influential predictors (Abrahamsson & Hyltenstam, 2008; Birdsong & Molis, 2001; Dörnyei & Skehan, 2003). In other words, under the same learning circumstances, differences in an individual’s initial state of readiness and capacity to acquire foreign languages, that is, their language aptitude (Carroll, 1981), will largely determine their ultimate attainment (Doughty, 2019). In our own understanding, language aptitude is at least partly genetically determined. Most researchers, however, emphasize that the concrete nature of language aptitude is yet to be determined through future research, and describe it as something between a stable, fixed trait (see evidence provided by Wells, 1986) and a plastic, malleable dynamic state (see discussions in DeKeyser, 2019, and Wen et al., 2017). Language aptitude is further believed to comprise a set of abilities, including language analytical abilities, phonetic coding ability, rote memory (Wen et al., 2017), and working memory (Wen, 2019). The most widely accepted models of language aptitude (summarized in Wen et al., 2017), namely Skehan’s cognitive “Processing Stages” (Skehan, 1998, 2002, 2016) and Robinson’s interactive and pragmatically oriented “Aptitude Complex Hypothesis” (P. Robinson, 2001, 2012), address several cognitive and environmental variables, but do not explicitly address the neurocognitive basis of their frameworks. Thus, it remains unclear which brain areas and networks are involved in language aptitude, to what extent they modulate language learning success, and how they develop, and hence rely on potentially innate and/or prenatal factors. Likewise, research on individual differences in second language learning has for a long time focused on observable differences in behaviour (see a recent summary in Kidd et al., 2018), although it is clear that these differences are influenced by domain-general abilities (e.g., intelligence, memory; Ellis, 2004), which are rooted in brain structure and function (Campbell & Tyler, 2018).

Generally, the brain areas most likely implicated in language aptitude are those that contribute centrally to language comprehension and production (Price, 2010, 2012), cognition, and, memory (e.g., see review by Biedroń, 2015). These are primarily left frontal and perisylvian regions (inferior frontal, temporal and inferior parietal; as presented in the model of Hickok & Poeppel, 2004; Petrides, 2014), with contributions of right homologous regions as well (Vigneau et al., 2011). In the past two decades, the number of studies exploring the neurobiological bases of language aptitude and individual differences in language learning has constantly increased. However, uncovering the neural basis of language aptitude requires addressing its development both through nature and nurture—its genetic basis, in utero influences, peri- and postnatal factors, as well as its further development from childhood to adolescence. Even if research with infants and children is scarce, studies on individual differences in language processing and learning on a more general level can provide vital insights into the neural basis of language aptitude.

We here provide a comprehensive review of research on the neurobiology of language aptitude and individual differences in language learning, and a first, preliminary neurocognitive model of language aptitude. First, we discuss previous research exploring differences in structural and functional connectivity, functional activation patterns, and brain morphology. Second, we discuss the nature and nurture of language aptitude, which serves as a basis for the following model. Third, we present our model and elaborate on the neurocognitive basis of language aptitude, as well as the neurobiological differences and changes associated with language learning and high aptitude. Last, we present future avenues for research.

## BACKGROUND

Only a few studies to date have investigated the neural underpinnings of language aptitude. Considerably more but still limited research has aimed to capture the heterogeneity of language processing across individuals as so-called “individual differences” in language learning, which are evident in all domains and components of the language system (Kidd et al., 2018; Yu & Zellou, 2019), and comprise neurophysiological, neuroanatomical, cognitive, and perceptual levels (P. C. M. Wong & Ettlinger, 2011). For the following presentation of previous research, we divided research studies according to whether they explored structural or functional underpinnings, but we emphasize the constant interaction of the two in our model presented later (see the section, A Neurocognitive Model of Language Aptitude).

### The Neurobiology of Language Aptitude

#### Brain structure as an indicator for high language learning abilities

Structural characteristics related to high language aptitude have been found in grey and white matter volumes of the left inferior parietal lobe (IPL), the auditory cortices, and the left inferior frontal cortex. A study by Reiterer et al. (2011) reported higher grey matter volumes in the left IPL and the left inferior frontal/motor compound in more gifted speech imitators during the imitation of an unknown language. Similarly, higher grey matter volumes and a stronger gyrification (i.e., a more frequent occurrence of multiple gyri) were found in the right auditory cortices of children and adults with high speech imitation skills and high overall language aptitude (including language analytical abilities, vocabulary learning, and phonetic coding ability) (Turker et al., 2017, 2019). In these two studies, possessing a single gyrus as opposed to multiple gyri in the right hemisphere was associated with low language aptitude scores. Recently, higher cortical thickness in Broca’s area (left inferior frontal gyrus/IFG) and the left posterior-medial frontal lobe were related to higher language analytic abilities in adults. The thicker the cortex was, the higher the language analytic ability scores were (Novén et al., 2019).

Investigating white matter connectivity between language-related brain areas, Xiang et al. (2012) reported that each of four administered language aptitude tasks was differentially related to the strength of different white matter tracts. Sound-symbol correspondence learning, for instance, could be best predicted by the interhemispheric connections between left and right posterior IFG. Structural connections in the left temporal pathway (connecting the left inferior frontal cortex and temporal areas) predicted grammatical inferencing abilities, while connections in the left parietal pathway (connecting the left anterior IFG and the IPL) predicted vocabulary learning and sound-symbol learning. In another study, higher fractional anisotropy of the left arcuate fascicle correlated with high speech imitation abilities, while higher volume of the posterior right arcuate fascicle correlated with low speech imitation ability (Vaquero et al., 2017). Conversely, the anterior segment of the right arcuate fascicle was linked to high grammatical analytical abilities in another study (Kepinska, Lakke, et al., 2017), meaning that speech imitation and analytical abilities could be differentially reliant on segments of the arcuate fascicle.

In sum, whereas the auditory cortex seems to be important for overall language aptitude and speech imitation, inferior frontal and motor areas might be particularly important for language analytic abilities and speech imitation. The role of the left IPL is potentially related to phonological processes rooted in the temporo-parietal junction; and different segments of the arcuate fascicle, both in the right and left hemisphere, seem to be related to specific skills associated with language aptitude. However, more research is needed to verify these preliminary results and specifically, to determine the role of right-hemispheric white matter fibre tracts.

#### Brain function as an indicator for high language learning abilities

Studies that investigated functional activation have reported divergent and partially contradicting results so far. Reiterer et al. (2011) found remarkable individual differences in how subjects employed left-hemispheric speech areas during speech imitation tasks. Those with high speech imitation ability showed decreased brain activation in left frontal and parietal areas. This supports the hypothesis that high neural efficiency is associated with more focal brain activation (Neubauer & Fink, 2009). Conversely, Hu et al. (2013) reported more widespread and higher functional activation in auditory perceptual and speech motor areas in learners with better pronunciation. In this study, subjects’ English pronunciation skills, as well as their phonetic coding ability, were assessed and related to brain activation during a speech imitation task. On the behavioural level, phonetic coding ability was strongly tied to pronunciation proficiency. On the neural level, brain activation during speech imitation differed significantly between those with high and low pronunciation proficiency. More widespread activation was also found in learners with high language analytic ability (Kepinska, de Rover, et al., 2017), who displayed greater activation in terms of magnitude and extent in the left IPL and the right temporal cortex. The involvement of the right hemisphere in high language learning abilities was confirmed in a later EEG study (Kepinska, Pereda, et al., 2017), where higher proficiency during artificial grammar learning was supported by stronger local synchronisation in the right hemisphere, combined with less mental effort in the learners with high language analytic ability (for further evidence that low aptitude speakers might have fewer cognitive resources and struggle with increasing cognitive load, see Antoniou & P. C. M. Wong, 2015).

To summarize, studies investigating language aptitude with neuroimaging have primarily assessed speech imitation ability, pronunciation, and language analytic ability. For speech imitation and pronunciation, left-hemispheric language and in particular speech motor and auditory areas seem to play a dominant role, whereas language analytical abilities seem to be more tied to left inferior frontal and right-hemispheric brain activation. The observed differences between these studies (more focal vs. more widespread activation) could reflect differences in assessment of skills (i.e., which tasks were used to determine high or low language learning status) and applied tasks outside and inside of the scanner.

### The Neurobiology of Individual Differences in Language Learning

Similarly to language aptitude, individual differences in language learning success present as differences in grey matter volume, functional activation and connectivity patterns, and even functional connectivity in language-related areas during rest (see the review by Li & Grant, 2016).

#### Brain structure and individual differences in language learning

Research on individual differences in language learning suggests that the morphology of the left IPL, IFG, and auditory cortices (especially, Heschl’s gyrus (HG)) is strongly related to language learning success. Longitudinal evidence suggests that increasing grey matter density in the left IPL predicts foreign language learning success (Della Rosa et al., 2013). Furthermore, there is evidence for the importance of the auditory cortices for various language learning-related processes and mechanisms. For instance, higher white matter density in the left auditory cortex was found to be significantly linked to novel speech sound learning (Golestani et al., 2002; see summary in Golestani, 2014). In the study by Golestani et al. (2007), differences in the gyrification of the left HG were also found between good and poor phonetic learners. Similarly, higher grey and white matter volumes in left and right HG also distinguished expert phoneticians from a control group (Golestani et al., 2011). The same study also reported a higher likelihood of two complete HGs in phoneticians. Apart from the auditory cortex, the left IFG, a hub for neural activities (Yang & Li, 2012), has been linked to success in artificial grammar learning (Flöel et al., 2009). In that study, white matter integrity in fibres arising from the left IFG, but not the right IFG, correlated with interindividual variability of grammar learning performance, hinting towards a role of that area for grammar rule extraction. Moreover, higher grey matter volumes in the left IFG were associated with an increase in proficiency during intensive foreign language learning, which is why the authors of that study (Stein et al., 2012) suggested that an individual’s amount of learning was reflected in brain structure changes regardless of absolute proficiency.

Other areas structurally relevant for L2 learning seem to include the anterior cingulate (Legault, Grant, et al., 2019), although differences in this study depended on the learning context as well (Legault, Fang, et al., 2019). Similarly, another study suggests that multilingualism is linked to grey matter volumes of the bilateral caudate, and an enlargement of the dorsal and anterior portions of the left caudate nucleus prelearning (Hervais-Adelman, Egorova, & Golestani, 2017).

Multiple fibre tracts in the human brain are involved in successful language processing and thus most probably in language learning. For instance, Dick and Tremblay (2012) differentiate between two dorsal (superior longitudinal fasciculus and arcuate fasciculus) and five ventral fibre pathways (see also discussions in Catani et al., 2005, and Glasser & Rilling, 2008). The linguistic model proposed by Hagoort (2014) includes a selection of these tracts, and he emphasizes the importance of the (subdivisions of the) arcuate fascicle, the inferior fronto-occipital fasciculus, and the frontal aslant tract for all linguistics tasks (for a different proposal, see Catani & Bambini, 2014). Concerning white matter fibre tracts and their relationship to individual differences, a single study found that fractional anisotropy of the left parieto-temporal pathway (dorsal fibre tract and part of the arcuate fascicle) was positively correlated with sound-to-word learning (F. C. K. Wong et al., 2011). In the same study, a ventral pathway involving the extreme capsule and the left inferior longitudinal fasciculus, on the other hand, mediated auditory comprehension.

In sum, grey and white matter differences in the auditory cortices have been reported to relate to speech sound learning, and studies have emphasized the involvement of the left IFG for language-specific individual differences, especially grammatical learning. Individual studies further suggest the involvement of left inferior parietal regions, the anterior cingulate cortex, the bilateral caudates, and the left parieto-temporal pathway for high language learning abilities.

#### Brain function and individual differences in language learning

Individual differences in brain function comprise differences in neural efficiency, neural adaptability, and functional synchronization (Prat et al., 2007, 2019; Prat, 2011; Prat & Just, 2011). Prat and colleagues reported differences in resting state brain rhythms (accounting for 26–60% of variance during intensive language learning; Prat et al., 2019); an increase of functional connectivity due to higher demands (i.e., an interaction between synchronization and adaptability); and generally greater efficiency and synchronization in several language-related areas in successful language learners (Prat et al., 2007; Prat & Just, 2011). Greater or optimized cortical processing efficiency has also been shown in polyglots (e.g., see Jouravlev et al., 2021) and second language learning experts (Reiterer, Berger, et al., 2005; Reiterer, Hemmelmann, et al., 2005). Reiterer et al. (2009) further found that the non-language students showed an increase in phase synchrony of the lower gamma frequency range, particularly in the right hemisphere. These significant increases involved right frontal and parietal regions: generally, the higher the proficiency level, the lower the synchronization density. Similarly, higher intrinsic functional connectivity within left posterior temporal areas (Chai et al., 2016) and increased global network efficiency with distinct network patterns (Sheppard et al., 2012) were found to be predictive of L2 word learning capacity.

With regard to temporal and inferior parietal areas, Veroude et al. (2010) and Assaneo et al. (2019) reported individual differences in implicit auditory/phonological learning mechanisms. In the latter study, subjects who implicitly aligned their own concurrent syllable production to a perceived syllable rate (termed “high synchronizers” by the authors) outperformed those who remained impervious to external rhythm during a word learning task. On the neural level, the high synchronizers showed a highly interconnected audio-motor network and better synchronization in left frontal areas (see also Poeppel & Assaneo, 2020). Further studies have shown that the degree and local efficiency of the left superior temporal gyrus (STG) is linked to sound-to-word learning performance, predicting future spoken language learning success (Deng et al., 2016). Moreover, pretest functional connectivity between the left insula/frontal operculum and the left superior temporal lobe predicted non-native sound contrast learning (Ventura-Campos et al., 2013). In a study by P. C. M. Wong, Perrachione, and Parrish (2007), learning to detect and use lexical pitch led to an increase in activation in the left posterior STG in the successful learners, who already showed higher activation in bilateral temporal areas and the right inferior temporal gyrus pretraining. The less successful learners, on the other hand, exhibited an increase in activation in the right STG and the right IFG, as well as prefrontal and medial frontal areas. With regard to the left IPL, Barbeau et al. (2017) reported learning-induced plasticity effects during intensive language learning. In their study, functional activation of the left IPL before the onset of training predicted posttraining attainment. Moreover, an increase in activation of the left IPL (specifically, the supramarginal gyrus) accompanied language learning, and higher activation in that region correlated with second language reading speed postlearning.

Studies have also provided evidence for a role of the left IFG in tonal vocabulary learning (Yang et al., 2015), lexical pitch learning (Qi et al., 2019), and statistical word segmentation learning (Karuza et al., 2013). More specifically, Yang et al. (2015) found that successful learners of Chinese showed distinct patterns in core language areas during tonal and lexical information processing and an overall more coherent and better integrated multipath brain network. Qi et al. (2019), on the other hand, found that greater pretraining activation of the right IFG was associated with better ultimate attainment. The key to success in these learners was greater pre- to postreduction of right IFG activation, coupled with enhanced resting-state connectivity between the right and left IFG and the left superior parietal lobe. Overall, learning was accompanied by increasing activation in left IFG and left superior parietal areas. Last, Karuza et al. (2013) reported significant activity during statistical word segmentation learning in pars opercularis and pars triangularis of the left inferior frontal cortex, and suggested potential parallels to the frontal/subcortical network involved in implicit sequence learning. Moreover, when progress is made during foreign language learning, shorter activation of left IFG occurs, which has been interpreted as a faster L2 processing (Stein et al., 2006).

Studies show that individual differences in brain function often present as greater efficiency and higher synchronization of language-related brain areas in both hemispheres. Specifically, learning success involves the functioning of left temporal, left parietal, and left inferior frontal areas. However, right-hemispheric areas seem to be involved in a multitude of tasks and in successful as well as nonsuccessful learners, which requires further research.

#### A potential role of subcortical structures for language learning

Individual differences in language learning have also been confirmed at subcortical levels, for example, in the inferior colliculus for the encoding of non-native phonemes (Chandrasekaran et al., 2012) or the left hippocampus for vocabulary acquisition due to its role in episodic learning (Breitenstein et al., 2005). As discussed later on, implicit and explicit learning systems are vital for foreign language learning (see a recent summary on the model proposed by Ullman, 2015). The findings that implicit, subcortical learning systems (e.g., in the basal ganglia; see review in Ullman, 2004) may be altered in individuals with developmental language disorders and dyslexia (Krishnan et al., 2016) suggest that these systems may play a significant role in various language-learning tasks in children and adults. This, in turn, could mean that language aptitude is highly dependent upon subcortical structures, which certainly requires future research. The same could be true for procedural learning, which is very likely to be implicated in rule-learning.

## THE NEUROCOGNITIVE BASIS OF LANGUAGE APTITUDE AND ITS HERITABILITY

### The Auditory Cortex and Its Role in Language Learning

From the current status of knowledge, the individual morphology of the human brain is at least partly genetically determined (Pol et al., 2006; Strike et al., 2015), although genetic influences on cortical morphology are a highly complex issue (Chen et al., 2013). Prenatal experience with speech shapes speech perception and production in newborns so that speech processing in newborns already displays a specialization for it (Gervain, 2015). Already in the second and third trimester of pregnancy, language-related areas show distinct patterns and asymmetries: Most sulci, including the Sylvian fissure, become visible between gestational weeks 21 to 27 (Bernard et al., 1988) and even individual gyral characteristics in structures like HG appear between gestational weeks 18 and 41 (López Ramón y Cajal, 2019). The primary cortical shapes and sulcal folding patterns are prenatally determined and under strong spatio-temporal genetic control (Chen et al., 2013; Thompson et al., 2001; for an in-depth review, see Im & Grant, 2019). While Bartley et al. (1997) reported that only 7–17% of gyral patterning of the entire brain appears to be due to genetic influences, studies with mono- and dizygotic twins have shown that the anatomy of HG is estimated to have a higher genetic determination (e.g., see Peper et al., 2007), with estimates of up to 80% (Pol et al., 2006); this genetic stability is supported by longitudinal behavioural and neural observations (Seither-Preisler et al., 2014). However, the specific genetic variants involved in HG morphology remain yet to be further determined (Cai et al., 2014) . It is assumed that the total variance in interindividual brain anatomy can be explained by (a) genetic, (b) in utero/prenatal, and (c) external postnatal factors (Carmelli et al., 2002). Dubois et al. (2010) reported large asymmetries in the superior temporal sulcus (also confirmed in Glasel et al., 2011), the planum temporale, and the anterior region of the Sylvian fissure (inferior frontal) in newborns from 26 to 36 weeks of gestation, providing evidence that the functional lateralization of language processing begins to manifest quite early.

The auditory cortex presents with large intra- and interindividual differences as revealed in early postmortem investigations (Auerbach, 1906; Heschl, 1878) and neuroimaging studies (Geschwind & Levitsky, 1968; Marie et al., 2016; Penhune et al., 1996; Rademacher et al., 2001; P. Schneider et al., 2002, 2005). These studies showed that HG appears as a single gyrus, a common stem duplication (partial division by a sulcus intermedius), a complete duplication (i.e., two complete gyri), or in the form of multiple gyri (Benner et al., 2017; da Costa et al., 2011). Duplications occur in every second or third individual (Marie et al., 2016) and the presence of multiple gyri has been linked to developmental conditions (Seither-Preisler et al., 2014), as well as to high musical ability (Benner et al., 2017; P. Schneider et al., 2005; Wengenroth et al., 2014).

Previous research on language-related skills (speech perception, sound learning) has shown the significant involvement of the bilateral auditory cortices (also concerning the number of gyri) in phonological processing, pitch pattern learning (P. C. M. Wong et al., 2008), and second language learning (Golestani et al., 2007, 2011; Ressel et al., 2012). These findings comprised both the structural and functional level. Ressel and colleagues found a significant correlation between larger grey matter volumes in left HG and bilingualism, indicating that bilingual language experience might alter grey matter volume in the auditory cortex (Ressel et al., 2012). In the study by Wong and colleagues (2008), less successful foreign pitch pattern learners possessed smaller grey and white matter volumes in the left auditory cortex only (not in the right, surprisingly). In their review, Wong and Ettlinger (2011) reported differences in lexical pitch learning on the neuroanatomical (larger HG volumes) and the neurofunctional level (higher bilateral activation). In the studies by Golestani and colleagues, higher white matter volumes in the left auditory cortices were associated with better foreign speech sound learning (Golestani et al., 2007). Moreover, they found a link between the occurrence of multiple and split HGs in the bilateral auditory cortices of expert phoneticians (Golestani et al., 2011). Similarly, in previous studies on language aptitude, the occurrence of multiple gyri and higher grey matter volumes of HG in the right hemisphere predicted high overall language aptitude and high speech imitation ability in adults and children, whereas the occurrence of single gyri was linked to low overall language aptitude (Turker et al., 2017, 2019). Golestani and colleagues suggested that the presence of morphological differences in the auditory cortex could be interpreted as an intermediate phenotype for auditory-related domain-specific aptitude (Golestani et al., 2011). The idea that auditory cortex morphology is predetermined and not a result of learning experience is further supported by several studies (e.g., Benner et al., 2017; Seither-Preisler et al., 2014; Serrallach et al., 2016). Seither-Preisler et al. (2014), for instance, found large interindividual differences in the grey matter volume of right HG, which were associated with musical aptitude. However, the longitudinal data revealed that musical training itself did not result in any neuroanatomical changes (those were only observed on the neurofunctional level of neural efficiency).

But why would auditory areas be so important for language aptitude? Auditory processing is the first capacity that develops in the fetus and the correct functioning of the auditory cortex is crucial for spoken language development (Mueller et al., 2012; Skeide & Friederici, 2016). Already during gestational weeks 28–33, the bilateral posterior STG shows mismatch responses to sounds and syllables, reflecting early auditory/phonetic abilities in the fetus (Mahmoudzadeh et al., 2013). Since there is a bias towards language-specific frequency spectra at that early developmental age, it is highly likely that primary, intrauterine speech perception is genetically driven (Skeide & Friederici, 2016). Soon after birth, the auditory areas are connected to the motor cortex through a myelinated fibre tract, which is crucial for developing phoneme representations in the brain (Dubois et al., 2010; Perani et al., 2011). The right secondary auditory cortex has been shown to respond to sentences, which might indicate a reliance on suprasegmental information (e.g., stress, melody, or intonation) during the first stage of language development (Homae et al., 2006). During months 6–12, infants are already capable of detecting phonological word forms, but at the same time perceptual narrowing takes place, meaning that an infant’s phonological system is established and their brain becomes specialized for the language they encounter daily (Pons et al., 2009). Few children, so-called “early talkers,” even show full-sentence highly developed verbal communication skills at 2 years of age. This likely reflects a proactive willingness to communicate and high intrinsic motivation to engage in language activities, which is claimed to be a marker for enhanced language aptitude (Gross, 1999; Winner, 1996).

### The Heritability of Language Aptitude

In the past years, researchers have started to identify the genes involved in speech and language, but we are still at the beginning (see the review by Fisher, 2017). Twin and heritability studies suggest that the rate of language acquisition and the linguistic proficiency attained by an individual are largely determined by genetic factors (Stromswold, 2001; Verhoef et al., 2020; see also discussion on the latter in Chow & S. W. Wong, 2021), which is supported by neuroscientific studies finding that second language learning correlates with white matter fibre tracts and genetic variation (Mamiya et al., 2016). With regard to general cognitive ability, heritability has been found to increase linearly from child- to adulthood (Plomin et al., 2016), with genetic influence accounting for 41% of individual differences in behaviour in 9-year-old children, 55% in 12-year-olds, and 66% in adolescents aged 17 (Haworth et al., 2010). Finally, up to 80% of the variance in full-scale IQ, also comprising language-related skills and general intelligence, can be accounted for by genetic variance components in adults (Plomin & Deary, 2015). Focusing on second/foreign language learning specifically, twin studies with children, teenagers, and young adults suggest moderate to high heritability estimates of 42–72% (42% in Dale et al., 2012; 67% in Dale et al., 2010; 71% in Vinkhuyzen et al., 2009; 72% in Coventry et al., 2012). Rimfeld et al. (2015) reported that all measures of second language learning showed high heritability (36–62%) even for different first languages and that one third of the genetic influence in second language learning was shared with intelligence. Targeting much younger children, Rice et al. (2018) calculated heritability estimates in the range of 44–92% in 6-year-old children, with the highest estimate being at 0.92 for grammar, and an increase in heritability from 4 to 6 years of age.

It seems very likely that adults will gain higher levels of foreign language mastery the higher their predetermined aptitude and the more prominent the neural perquisites and changes. These conclusions are supported by studies that found that native-like proficiency in foreign language learning is rare and most likely a result of language aptitude, not age of onset (see Abrahamsson & Hyltenstam, 2008, 2009). Although inherited genetic information may set limits on what can be achieved by an individual, it is the environment that determines what individuals actually accomplish (Dörnyei, 2014; for a discussion on gene-environment interactions in language learning, see Onnis et al., 2018). In the past decade, the emerging field of epigenetics has allowed valuable new insights into the interplay between nature and nurture, also on the neural level (e.g., refer to Dehaene-Lambertz et al., 2006). It has shown not only that genes have an effect on learning efficiency and general cognitive abilities, but also that learning experience has a reciprocal effect on gene expression (Bjorklund & Causey, 2018), which is particularly interesting for education (Mc Ewen, 2015).

Overall, it seems that language-related abilities, such as language aptitude, are highly heritable and genetics might exert a larger influence at a later age. However, more research is needed to pinpoint the genetic underpinnings of language, and further describe gene-environment interactions with regard to language.

### The Role of Cognitive Abilities, Musicality, and Memory During Language Learning

Cognitive abilities develop and come into play at different stages of language learning. Since hearing develops in the fetus long before birth (around gestational week 27; Hepper & Shahidullah, 1994), it is not surprising that fetuses already show mismatch responses to unexpected sounds and tones (Huotilainen et al., 2005). Interestingly, even in that early stage slight individual differences have been observed (Draganova et al., 2005). One-year-old infants have been shown to possess working memory in the visual (Ross-Sheehy et al., 2003) and auditory modality (Ross-Sheehy et al., 2003), but still show clear capacity limits. Concerning other cognitive capacities, Demetriou et al. (2014) postulated that fluid intelligence develops in four reconceptualization cycles between 2 and 16 years of age and suggested that working memory and processing speed are vital for fluid intelligence development. While a strong link between the three is likely, other researchers have proposed that the developmental increases in working memory arise primarily from improvements in other cognitive domains, such as attention, encoding, processing speed, and retrieval (Towse & Hitch, 2007). Most likely, working memory, the portal to long-term memory, underpins cognitive processes (Cowan, 2014), and largely interacts with learning efficiency and fluid reasoning (see discussion of the specific cognitive components that are essential for language learning in the section, Environmental Influences and Gene-Environment Interactions).

Musicality has also been shown to impact language learning (see recent review on interactions between language aptitude and music on the neural level by Turker & Reiterer, 2021). In the early stages of language learning, infants are acutely sensitive to prosodic patterns, which enables later phonological development (Gervain & Mehler, 2010). This suggests that this quasi-musical auditory structuring ability (Karma, 1994) can be advantageous for language learning. In line with our assumption that cognitive abilities are largely genetically driven, high musicality in the form of better rhythmic and melodic perception could be well linked to language aptitude at the genetic level. Recent genomic studies, for instance, have hinted towards a link between musical aptitude-related genes and auditory perception, cognitive performance, memory, and language acquisition (see Järvelä, 2018, for a review). As such, language and music would directly impact one another, but both would unfold and develop side by side, making a classical one-before-the-other-distinction unnecessary. This could perhaps take the form of an overarching compound of speech-music abilities, tied together by auditory and fine motor abilities with a common root. As such, advanced musical processing abilities could lead to better language perception, which in turn would result in higher musical discrimination abilities (Bowles et al., 2016; Delogu et al., 2008; Nardo & Reiterer, 2009; Schön et al., 2004). This could explain the frequent co-occurrence of speech imitation talent and musicality in adult language learners (Christiner & Reiterer, 2013; Nardo & Reiterer, 2009) even as early as in 4–6 year old children (Christiner & Reiterer, 2018). Moreover, research has further pointed towards a link between musical experience and higher success during the learning of phonetic aspects of languages (e.g., perception of pitch patterns; P. C. M. Wong & Perrachione, 2007), potentially due to their advantage in the discrimination of musical features, like tones.

It remains to be uncovered how (and when) declarative and procedural memory, two domain-general, cognitive, long-term memory storage systems, are related to foreign language learning success. Overall, it seems that declarative memory could be highly relevant in early L2 learning stages, and procedural memory in later learning stages (e.g., Faretta-Stutenberg & Morgan-Short, 2018). Furthermore, another study found that declarative memory was more relevant in implicit, exposure-based learning circumstances, while procedural memory was vital during incidental, immersive context-based learning (Antoniou et al., 2016) (see a thorough discussion in Buffington & Morgan-Short, 2019). Recently, Hamrick et al. (2018) provided clear evidence for a significant role of general-purpose learning systems in both first and foreign language acquisition in several meta-analyses. Overall, they reported that lexical ability was associated with declarative learning in all learners, while grammar was linked to declarative memory in low ability second language learners, and to procedural learning in the highly competent learners. Neurally, Morgan-Short and colleagues (Morgan-Short et al., 2014, 2015) found that some learners used the neural circuits of their first language and their procedural memory circuits when implicitly learning an L2, while others engaged extralinguistic neural circuits. It has also been suggested that procedural memory shares neural substrates with grammar learning (e.g., Broca’s area and basal ganglia; Ullman, 2004), since procedural memory has been associated with performance for simple words during a morphophonological grammar learning task (Antoniou et al., 2016; Ettlinger et al., 2014). Declarative and procedural learning, alongside working memory, could therefore be central ingredients to language aptitude. Considering these developments together with overall language learning, it becomes clear that all are essential prerequisites of first language acquisition, which in turn shows large long-term crosslinguistic transfers to foreign language learning (Sparks, 2012; Sparks et al., 2009).

### Neural Plasticity and Language Aptitude

Learning, the basis of intelligent behaviour, is caused by plastic changes in neural assemblies (Partanen et al., 2013). As Zhang and Wang (2007) summarize, learning-induced plasticity can present as (1) higher neural sensitivity, (2) increased neural specificity (i.e., a process-specific specialization of regions/pathways; Johnsrude et al., 2000), (3) stronger neural connections, and (4) enhanced neural efficiency (which may include changes in (1) to (3) at the same time, leading to faster and shorter activation; as found in Zhang et al., 2005). However, the concept of neural efficiency is still debated, and how the brain adaptively reallocates its resources is controversial. Additionally, learning-specific changes and enhancements are hard to separate from changes in attentional, cognitive, and memory-related processes (see discussion in Zhang & Wang, 2007).

The auditory cortex, which is a major region involved in language processing, shows perceptual narrowing during specific sensitive periods early on (Ortiz-Mantilla et al., 2016). Auditory learning leads to the formation and strengthening of long-term memory traces, which in turn positively influence discrimination skills that are the basis of speech perception and comprehension (Partanen et al., 2013). Hence, infants learn very early how to encode auditory features in the primary auditory cortex, group speech sounds they perceive into language-specific phonetic categories, and process musical rhythms and harmonic relationships (White et al., 2013). The auditory cortex is likely to have an extended period of heightened developmental plasticity throughout childhood, where changes in cellular organization occur (Kral & Eggermont, 2007). It is thought that during such sensitive periods (i.e., epochs during which experiences cause enhanced, long-lasting effects on behaviour and the brain; Penhune, 2011), neural representations are first broadly tuned and subsequently become more refined and respond preferentially to more frequently encountered features and stimuli in the environment (Scott et al., 2007). With regard to language learning, however, it seems that the neural systems involved therein are first established for optimal processing of the first language (frequently encountered input), and have to be adapted for the successful and efficient processing of another language (White et al., 2013).

Nonetheless, a major question concerns the interaction between experiential and maturational factors that either restrict or facilitate language learning across the lifespan. Regardless of environmental stimulation and extrinsic motivation, the potential for successful late L2 acquisition is significantly reduced on the neural level due to maturational declines in synaptic density, decreased levels of brain metabolism (Bates et al., 1992), and increased axon myelination (Pulvermüller & Schumann, 1994). Concerning explicit learning mechanisms, it might be the case that purely bottom-up (implicit) learning is not sufficient for later learners to change the phonetic representations built during L1 learning (Archila-Suerte et al., 2012). On the other hand, goal-oriented explicit training (i.e., progressive adaptation to performance, feedback, and directed attention to relevant features of the new language) may enhance post-sensitive period L2 learning (White et al., 2013).

Overall, much more research is needed to confirm or potentially help improve current models on language learning. A major problem in designing and interpreting studies is to further disentangle the influence of cognitive, memory-related, and attention-related processes and their impact on neural resources. Overall, increases and enhancements in neural specificity, efficiency, sensitivity, and connectivity are the basis of learning-induced changes in the brain.

## A NEUROCOGNITIVE MODEL OF LANGUAGE APTITUDE

**Figure 1.**
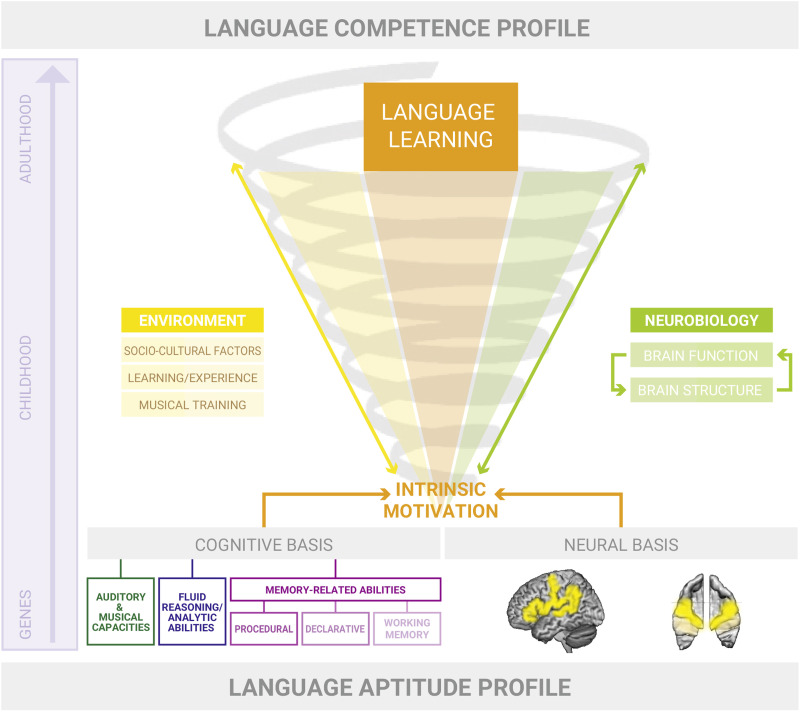
A neurocognitive model of language aptitude. Our model proposes that a specific, largely genetically and prenatally predetermined language aptitude profile progressively develops into a language competence profile. The language aptitude profile comprises a set of cognitive predispositions for language learning and its neural basis, which is visible as anatomical characteristics in the auditory cortex and other language-related regions (e.g., left IPL or IFG). In our model, advantageous neurocognitive predispositions foster intrinsic motivation and thus a general interest in language learning. Environmental factors (yellow; left side of the spiral) such as sociocultural factors, learning/experience, and musical training significantly contribute to the language learning process and interact with it on all levels. During development from early infancy into adulthood, the brain undergoes major neuroplastic changes that are partly biologically induced (maturational aspect) and partly learning-induced (biographical aspect). The extent of learning-induced neural plasticity is a function of the predisposed language aptitude profile (neural characteristics and its cognitive correlates) and supportive environmental factors.

Based on previous findings, we here present a preliminary neurocognitive model of language aptitude (see Figure 1). In our model, language aptitude is treated as an overarching ability based on neural and cognitive characteristics that are partly biologically determined and unfold and develop in interaction with the social environment. The model is an array of ideas based on previous research (including our own) and comprises two major profiles, namely a language aptitude profile and a language competence profile (in analogy to Seither-Preisler et al., 2014). Over time, due to biological maturation processes and in interaction with the environment (e.g., sociocultural factors, education, language/learning experience, musical training), the initial aptitude profile develops into a manifest competence profile (in accordance with the competence level proposed in models by Gagné, 2004). In our model, we assume that genetic attributes influence how individuals experience and interpret their environment, that is these attributes fundamentally guide the selection, modification, and creation of experiences in an individual, thus steadily complementing their competence profiles (Bjorklund & Causey, 2018; Scarr & McCartney, 1983). As such, an advantageous innate, or congenital, aptitude profile leads to a positive attitude towards language learning, which in turn fosters intrinsic motivation and eagerness to engage in language learning activities (as proposed in the socioeducational model of second language learning by Gardner, 2010). Previous research has shown that learning the full set of grammatical rules of one’s first language may take up to 17 years (Hartshorne et al., 2018), which is why we emphasize the fluent transition of all learning stages and from first to second and further foreign language learning. We further suggest that the individual amount of neural plasticity, which determines the speed and success of language learning, is proportional to language-relevant predispositions in the aptitude profile, which are determined by genetic, epigenetic, and intrauterine factors prior to birth. Although there is no doubt that the cellular mechanisms underlying experience-dependent structural changes in the human brain are crucial as well (for a summary, see Zatorre et al., 2012), the focus of the present model shall lie on stable morphological characteristics of cortical regions.

### The Language Aptitude Profile

#### The cognitive basis

In our view, the cognitive basis of language aptitude comprises the set of abilities/capacities that are essential for facilitating later language learning and that an individual possesses either before prenatal influences come into play (genetically predisposed), or that develop in early prenatal stages (influenced by genetical predispositions and intrauterine factors). The cognitive basis of our model is influenced by the Cattell-Horn-Carroll (CHC) model of human cognitive performance and intellectual ability (Carroll, 1993; Horn & Cattell, 1966; J. W. Schneider & McGrew, 2018) and is intricately tied to language aptitude. In our view, it comprises (1) auditory and musical processing, (2) fluid reasoning/analytical abilities, (3) memory-related abilities, such as (a) procedural memory/learning (e.g., learning efficiency), (b) declarative memory/learning (e.g., comprehension-knowledge), and (c) working memory.

Auditory processing, as suggested in the CHC model, comprises basic auditory hearing functions, language-specific abilities (phonetic coding, speech sound discrimination, resistance to auditory stimulus distortion, hearing/speech threshold), and music-specific abilities (processing of pitch, timbre, musical intervals, harmonies, melodies, rhythms). We therefore subsume them under auditory and musical processing. These higher language- and music-relevant auditory functions represent aspects of auditory scene analysis, that is, the ability to group auditory stimuli relative to background noise in a meaningful way on the basis of spectral and temporal acoustic parameters (for a definition, see Bregmann, 1990, 2015). Fluid reasoning designates the ability to perform mental operations, and comprises induction, sequential reasoning, and quantitative reasoning (J. W. Schneider & McGrew, 2018). Thus, it is not only vital for intelligence and general cognitive mechanisms, but also for learning a language, especially for language analytic abilities. Due to the strong link between fluid reasoning and analytical thinking, we have subsumed the two in one category.

The first memory-related ability is procedural memory/learning, which includes the concept of learning efficiency (see also a discussion on procedural memory underlying learning in Ullman, 2015, 2016). According to the CHC model (J. W. Schneider & McGrew, 2018), learning efficiency describes an individual’s capacity to learn, store, and consolidate new information, comprising primarily associative memory. Conceptually, learning efficiency is strongly tied to fluid reasoning and is reliant on working memory, both visuo-spatial and auditory working memory (Wen, 2019). The second ability, namely declarative memory/learning (see Ullman, 2015, 2016 for a distinction between declarative and procedural memory), includes comprehension-knowledge, which is defined as a memory base built for continuously incorporating and communicating cultural knowledge, including language (associated with an often-neglected aspect of language aptitude, namely pragmatic language aptitude; Sedaghatgoftar et al., 2019). Less likely to play a role at the initial stages of language learning (an observation we made in our own studies that was already proposed in the model by Ullman, 2004, 2015, 2016), declarative memory becomes greatly important after the onset of first language acquisition. It includes metalevel abilities such as lexical knowledge, listening ability, communication ability, and grammatical sensitivity, which have been considered a part of language aptitude since the early models of Carroll (Carroll, 1981; Wen et al., 2017). There has been an ongoing discussion on how to best integrate working memory in a model of language aptitude, and in the present model, we have decided to include it as a memory-related ability in the largely predetermined aptitude profile.

#### The neural basis

In the present model, the starting point of language aptitude is the auditory cortex (HG), which in previous studies was found to be a neuroanatomical marker for individual differences in speech sound learning and phonetic experience (Golestani et al., 2002, 2007, 2011), language aptitude (Turker et al., 2017, 2019; see also discussion in Turker & Reiterer, 2021), and musical aptitude (Benner et al., 2017; Seither-Preisler et al., 2014). Since interindividual variation of the auditory cortex can even be observed at a prenatal stage, this cortical structure bears the potential to boost auditory processing for speech and language learning from very early periods on. As such, these differences in morphometry could foster and aid an early and efficient development of cortical connections between auditory and motor areas (Skeide & Friederici, 2016). These auditory-motor connections are the basis of an efficient neural analysis of speech sounds, and thus of language processing (Möttönen et al., 2013). Auditory-motor connections can lead to strong speech-motor association networks (i.e., strongly and reciprocally interconnected neural networks; e.g., Pulvermüller & Fadiga, 2010; Watkins & Paus, 2004) in left superior temporal, temporo-parietal, and frontal areas, also sometimes referred to as Hebbian learning circuits (Hebb, 1949). It is very likely that in the case of high language aptitude, these highly specialized functional units develop earlier, faster, and more efficiently through language learning experience.

The neurofunctional efficiency of the auditory cortex seems to be both a function of preexisting neuroanatomical traits and of explicit auditory training (Seither-Preisler et al., 2014; Serrallach et al., 2016), thus revealing the direct interaction of nature and nature on the level of the auditory cortex. As such, we suggest that individual structural variation in left and right auditory cortices accounts for individual variation in language learning, and thus language aptitude, which in turn significantly influences all later language learning processes. In addition, we assume that structural variation in other left-hemispheric perisylvian areas (e.g., left IPL), less researched in research to date, could be related to language aptitude as well (see bottom right of Figure 1, where the language areas in the left hemisphere are marked in yellow). However, the relative contributions of predisposition and experience in this case need to be further researched, since only limited research has been conducted to date (e.g., see Hervais-Adelman, Moser-Mercer, et al., 2017; Stein et al., 2012).

As earlier mentioned, genes influence (social) behaviour (G. E. Robinson et al., 2008). Consequently, genetic propensities potentially guide children to individually select, modify, and create their experiences (see further evidence in Plomin, 1994). We argue that children and adolescents with an advantageous language aptitude profile will feel particularly motivated to learn foreign languages due to the ease with which they acquire them and the progress they make without substantial effort (Carroll, 1981). The above-described facility for learning languages is likely to result in primarily positive associations with language learning and heightened metalinguistic awareness (Jessner, 2008, 2014), which in turn facilitates future language learning through higher comprehension-knowledge and learning efficiency (i.e., both declarative and procedural learning would be affected) within this domain. According to our model, congenital abilities and acquired skills (nature-driven and nurture-enhanced processes) constantly interact with one another, evolve, reinforce, and influence one another in a spiral-like fashion or like moving upwards/downwards in a spiral (which is displayed in Figure 1, where all components of the spiral, because of their interconnectedness, influence the top and bottom of the spiral continuously and at the same time). If a lot of experience or training falls on enhanced grounds that is based upon better audio-lingual starter conditions, exceptional skills can be expected. On the other hand, untrained or barely trained positive starting conditions will not be enough to develop high language competence, as in the opposite case of less gifted learners. In the absence or with a very low level of auditory-language abilities (e.g., language impairments), even high levels of training will not easily compensate and bring forth high competence levels. Only with sufficient effort and time, individuals with low aptitude can learn and improve their language skills.

### Environmental Influences and Gene-Environment Interactions

In our view, the three environmental variables that most significantly influence language learning are (1) sociocultural and socioeconomic factors, which are particularly relevant during early language learning in childhood, (2) previous (language) learning experience (including the quality and quantity of language input), and (3) musical training. The first includes all variables that relate to both the social and cultural background and the socioeconomic environment of an individual (including socioeconomic status, parenting style, parent-child interaction, and education) and that shape early language learning experiences (Hayiou-Thomas, 2008; Hoff, 2006). At the same time, research has shown that the neural circuits of language are shaped by previous language learning experience (Pierce et al., 2015; Sharpe et al., 2021), which supports our suggestion that any form of learning experience either directly or indirectly guides and influences future learning situations (e.g., by exerting a direct influence on intrinsic motivation. Behaviourally, first language learning difficulties, reading deficits, or potentially negative experiences in foreign language learning classes, coupled with anxiety, negative feedback, and a loss in motivational intensity, can negatively impact future foreign language learning (see discussion in Ellis, 2004; Fonseca-Mora & Machancoses, 2016). As such, the quality and quantity of the language experience will exert an influence on long-term second language learning attainment (see also proposal of Moyer, 2008). While we do not propose a specific model for language learning experience, we believe that language learning closely matches general learning patterns and mechanisms, which are thought to rely on attention, the enhancement of expectancies, and autonomy, which largely influence intrinsic motivation (for an interesting proposal on motor learning, see Wulf & Lewthwaite, 2016).

A third essential environmental variable, namely musical training, influences language learning on the neural level, as well as on the cognitive level (Turker & Reiterer, 2021). From a neural perspective, both language and music recruit an array of brain networks involving motor, auditory, visual, and memory-related mechanisms. Auditory processing at subcortical and cortical levels benefits from musical training, leading to stronger brainstem responses (e.g., to pitch; Kraus & Chandrasekaran, 2010; Moreno & Bidelman, 2014; P. C. M. Wong, Skoe, et al., 2007) and faster and bilaterally more synchronous cortical auditory evoked responses (Seither-Preisler et al., 2014; Serrallach et al., 2016). Musicianship has further been shown to enhance speech perception, linguistic skills, and high-level cognitive processing (Moreno et al., 2011; Schellenberg & Winner, 2011), leading to positive and long-lasting benefits on auditory functioning. The inextricable link between language and music (Patel, 2012; Schellenberg & Peretz, 2008) has even led researchers to explore whether precursors of the two domains have evolved together or from a common signal system (e.g., “musilanguage” in Brown, 2000). In any case, it should be expected that both domains are strongly related on a neuropsychological level, an assumption that has been corroborated in a multitude of studies (Sammler, 2020).

### Preliminary Hypotheses and Predictions

The current model suggests that a specific language aptitude profile may strongly impact the level of intrinsic motivation. As such, an advantageous profile would lead to a heightened motivational intensity, which in turn fosters the language learning process (Gardner, 2010). While intrinsic motivation is displayed as the basis of all language learning, we emphasize that intrinsic motivation is presumably more involved in language learning processes other than first language learning, which is most likely largely driven by the biological need to communicate within the social environment. It thus seems meaningful to discuss the particular neurobiological changes and mechanisms related to the unfolding of language aptitude separate from the concrete interaction of environmental variables with cognition and language learning.

The neural properties of the linguistically gifted brain could manifest in various ways, from (partly) innate morphological differences to thicker myelination around language-related fibre tracts to higher efficiency during linguistic tasks. Such efficiency could manifest as more focal activation in tasks requiring convergent thinking, or more widespread activation in tasks requiring divergent thinking. We have formulated some hypotheses regarding brain function and structure, and their relation to the development of a language aptitude profile into a competence profile, as displayed in Figure 1.

#### Neurofunctional underpinnings of language aptitude

As discussed earlier, only a few studies to date have directly related functional brain activation to language aptitude, and so far, these have yielded mixed results. Studies have reported either more focal or more widespread neural processing indicative of high language talent and competence (Hu et al., 2013; Kepinska, de Rover, et al., 2017; Reiterer et al., 2011). Although it is not self-evident how both observations can be true at the same time, they are not mutually exclusive. First, these discrepant results could be consequences of task choice, meaning that artificial grammar learning (grammatical analytical task), being a more complex and cognitive task compared to speech imitation, could require more widespread activation and it might thus be harder to develop more efficient processing. At the same time, however, Kepinska, de Rover, et al. (2017) reported only differences between high and average grammatical ability learners since they found no differences between high and low ability learners. This might stem from other limitations of that study (sample size, specific task choice, characteristics of the learner groups). Another explanation for the differences in results could be the learning stage or level (beginner vs. advanced).

We agree with Prat et al. (2019), who suggests that individual differences in language learning, including high language learning ability, manifest in neural efficiency, neural synchronization, and neural adaptability. Concerning brain activation, we therefore hypothesize that brain activation should be more focal in high ability learners compared to low ability learners due to previous learning experience and expertise in the specific language-related tasks. This should hold true for all domains of language learning, from pronunciation and sound learning to grammatical analytic abilities. While we believe that studying differences in functional activation within language-specific brain areas certainly provides interesting results as to the engagement of relevant areas, they can only provide us with a short glimpse into how functioning machinery can look. If there are distinct functional brain networks for speech articulation, sensory language processing, and higher-level language processing, as suggested by Fedorenko and Thompson-Schill (2014), individual differences related to language aptitude need to be investigated in these networks. Previous investigations on differences in functional and effective connectivity, absent in language aptitude research but extensively studied regarding individual differences in language learning, portray a much clearer picture. In highly successful language learners, a variety of language- and domain-general areas in the human brain have been found to work in concert and show greater functional and effective connectivity, often in the absence of clear functional activation patterns (e.g., Prat et al., 2019). In particular, connectivity of the left IFG and left STG seem to be frequently observed within language learning tasks. We propose that more efficient global language networks, be it during rest or during/after extensive foreign language learning, could be indicators of and at the same time the result of language aptitude. In accordance with our view that the posteromedial HG and the posterior STG, hosting primary and secondary auditory areas, are crucial for language aptitude, we see functional connectivity differences at rest or during linguistic processes as potential indicators that successful language learning is the result of well-myelinated connections between these auditory structures and inferior frontal areas.

#### Structural underpinnings of language aptitude

Evidence so far suggests that structural characteristics of the bilateral auditory cortices (Golestani, 2014; Golestani et al., 2002, 2007, 2011; Turker et al., 2017, 2019) and the left inferior frontal cortex (Novén et al., 2019) are involved in high language aptitude. Moreover, portions of the left and right arcuate fascicle have been found to relate to language aptitude as well (Kepinska, Lakke, et al., 2017; Vaquero et al., 2017; Xiang et al., 2012). Consequently, we suggest that specific structural characteristics (e.g., higher grey matter and higher gyrification) should be found in individuals with high language learning ability. Moreover, we believe that learning experiences will lead to faster changes in these areas (i.e., individuals with higher language learning abilities will show faster response to training and possess a heightened neural plasticity).

Regarding structural connectivity, we would like to elaborate on ideas related to the maturation of the arcuate fascicle and how it could foster language learning. Language accuracy and processing speed have been shown to depend upon the maturational status of the arcuate fascicle (Skeide et al., 2016). Fractional anisotropy, and other maturational indicators, for example, myelination (Mukherjee et al., 2001), axon growth (Paus, 2010), and increasing fibre density (Scholz et al., 2009), are essential for the refinement of the dorsal syntax network. This dorsal pathway from left temporal areas to posterior Broca’s area (left IFG) is important for higher-order language functions since it is weak in nonhuman primates and weaker in children than in adults (Friederici, 2009). Learning a new or an artificial grammar is closely tied to sentence-level syntax, which is primarily driven by the left IFG modulating the posterior STG (Makuuchi & Friederici, 2013), and syntactic information is exchanged dorsally along the arcuate fascicle (Friederici et al., 2011; Saur et al., 2008). However, the two dorsal fibre tracts that interconnect left temporal and frontal areas in adults do not develop simultaneously in infants. Only the fibre tract to the premotor cortex, thought to be responsible for the integration of sensory and motor representations during babbling and the development of phonemic representations, is already myelinated in infants of 2–5 months of age (Dubois et al., 2010; Perani et al., 2011). We thus hypothesize that an earlier maturation of the left arcuate fascicle, as well as a stronger left lateralization of specific segments of this fibre tract (specifically, the left posterior segment) could be a marker for high language aptitude. More importantly, higher myelination of that fibre tract could be a direct indicator for faster processing between posterior STG and IFG, which should result in more efficient communication between these areas and thus likely reflects high language learning abilities.

Apart from the arcuate fascicle, we hypothesize that the frontal aslant tract (pronunciation and imitation), the uncinate fasciculus (potentially involved in memory retrieval and learning efficiency), and the inferior fronto-occipital longitudinal fasciculus (phonetic coding, phonological processes, as well as semantic-related learning) could be key tracts to further investigate with respect to language aptitude.

#### Experience- and learning-dependent plasticity effects

Although grey and white matter volumes in most areas of the brain increase in early childhood, declines in frontal, parietal, and temporal areas both in terms of volume and thickness have been reported across adolescence (Foulkes & Blakemore, 2018). What should not be forgotten, however, is that these primarily group-level findings may be subject to considerable interindividual differences, resulting from both the genes and the environment. With regard to language learning, a plethora of studies have reported differences in neural plasticity (e.g., cortical thickness) related to various forms of language learning, for example, due to language learning onset (Vaquero et al., 2020), novel word learning (Shtyrov et al., 2010), or intense language learning studies (Mårtensson et al., 2012). So far, very little overlap regarding the brain regions affected by cortical thinning/thickening has been reported. Leading to more confusion, changes due to learning have been shown in numerous studies (e.g., Zhang et al., 2009, reporting brain activation changes due to phonetic learning in adulthood) but little is known about whether these changes pertain and lead to life-long modifications or are just temporarily built during intensive learning phases.

It is widely accepted that learning, regardless of whether it is of linguistic nature, leads to a modification of biochemical processes (at the subcortical and cortical level through a modulation of synaptic plasticity; Sharpe et al., 2021). Such changes due to learning occur in all individuals, regardless of their specific capacity for learning and often even irrespective of the learning period and intensity. Although it seems logical to assume that neuroplasticity is more a sign of learning than of language aptitude per se, it is likely that neuroplastic changes happen faster and are more effective in those with high language aptitude. Moreover, research suggests that the amount of learning-induced neuroplasticity is directly proportional to biologically predisposed aptitude (Seither-Preisler et al., 2014; Serrallach et al., 2016), which, however, requires more research.

## CONCLUSIONS AND FUTURE DIRECTIONS

Despite the substantial improvements in research on individual differences and cognitive capacities, very few studies to date have explicitly tackled the investigation of language aptitude and its relation to neurobiological patterns and mechanisms. Studies with only adults provide a short and limited glimpse into already established, functioning machinery, and so far, language aptitude and individual differences have only been investigated in spoken languages, whereas sign languages have been completely left out. Certainly, uncovering the neural bases of language aptitude, be it in the spoken, auditory, or signed domain, requires addressing the development of language aptitude both through nature and nurture in utero, and in peri- and postnatal stages and up to childhood and adolescence. The ideas we present in this model are based on spoken language mainly and cannot account for signed languages yet.

As mentioned in the abstract, the model presented here is preliminary and shall be refined as more research on the topic emerges. To confirm the above formulated hypotheses, further behavioural and neurobiological longitudinal and molecular genetic studies are desperately needed, especially with young children and adolescents. Other avenues for future research include investigating relative contributions of experience-dependent plasticity, especially in contrast to potentially preexisting indicators for learning and plasticity (e.g., domain-specific aptitude; Golestani, 2014; Golestani et al., 2011). Additionally, we suggest that future functional neuroimaging studies should investigate the involvement of functionally specialized and domain-general language-related networks to explore language aptitude on a network level (Fedorenko & Thompson-Schill, 2014). Additionally, even if the arcuate fascicle is one of the most prominent fibre tracts for higher-level language processing and cognition, we propose to further investigate other fibre tracts in relation to specific language subskills, for example, the frontal aslant tract for speech imitation and pronunciation proficiency, the uncinate fasciculus for semantic-related memory retrieval (e.g., necessary in vocabulary learning), or the inferior fronto-occipital fasciculus for specific phonetic and phonology-related skills. In the future it might also be worthwhile to incorporate intergenerational genetic transmission patterns and their variation in men and women, for example, as has been shown for corticolimbic circuity (Yamagata et al., 2016), which would add an additional perspective to the present model.

## FUNDING INFORMATION

Sabrina Turker, Alexander von Humboldt-Stiftung (https://dx.doi.org/10.13039/100005156), Award ID: Postdoc Fellowship.

## AUTHOR CONTRIBUTIONS

**Sabrina Turker**: Writing – original draft: Equal; Writing – review & editing: Equal. **Annemarie Seither-Preisler**: Writing – original draft: Equal; Writing – review & editing: Equal. **Susanne Maria Reiterer**: Writing – original draft: Equal; Writing – review & editing: Equal.

## TECHNICAL TERMS

Language aptitude: An individual’s (largely innate) capacity for acquiring foreign languages without much effort and faster as compared to peers.
Phonetic coding ability: A component of language aptitude that includes the identification of sounds, the formation of associations between sounds and letters, and the ability to retain these associations.
Language analytic abilities: A component of language aptitude that designates the ability to analyse language (mostly explicitly) and arrive at linguistic generalizations (i.e., draw conclusions and infer rules and regularities).
Sound-symbol learning: One part of the LLAMA language aptitude battery in which subjects have to learn a new sound-symbol system (i.e., build associations between new linguistic units and their corresponding auditory form).
Arcuate fascicle: The major language-related white matter fibre tract that connects temporal areas with frontal areas via the inferior parietal lobe.
Learning efficiency: An individual’s capacity to learn, store, and consolidate new information, comprising primarily associative memory in the CHC model.
Fluid reasoning: The ability to perform non-automatic mental operations comprising induction, sequential reasoning, and quantitative reasoning.
Learning-induced neural plasticity: Changes on the neural level caused by learning processes (e.g., learning a new language).
Cattell-Horn-Carroll (CHC) model: A model of human cognitive performance and intellectual ability.
Auditory scene analysis: The ability to group auditory stimuli relative to background noise in a meaningful way on the basis of spectral and temporal acoustic parameters.
Comprehension-knowledge: A memory base built for continuously incorporating and communicating cultural knowledge, including language in the CHC model.
